# Facing Death: Attitudes toward Physician-Assisted End of Life among Physicians Working at a Tertiary-Care-Hospital in Israel

**DOI:** 10.3390/ijerph18126396

**Published:** 2021-06-13

**Authors:** Keren Dopelt, Dganit Cohen, Einat Amar-Krispel, Nadav Davidovitch, Paul Barach

**Affiliations:** 1 Department of Public Health, Ashkelon Academic College, Ashkelon 78211, Israel; 2 Department of Health Policy and Management, School of Public Health, Faculty of Health Sciences, Ben-Gurion University of the Negev, Beer-Sheva 84105, Israel; eynatam@post.bgu.ac.il (E.A.-K.); nadavd@bgu.ac.il (N.D.); 3 Cardiology Department, Barzilai University Medical Center, Ashkelon 7830604, Israel; dganitc@bmc.gov.il; 4 Pharmacology Department, Soroka University Medical Center, Beer-Sheva 84101, Israel; 5 Department of Pediatrics, School of Medicine, Wayne State University, Detroit, MI 48202, USA; Paul.Barach@jefferson.edu; 6 Jefferson College of Population Health, Thomas Jefferson University, Philadelphia, PA 19107, USA; 7 Interdisciplinary Research Institute for Health Law and Science, Sigmund Freud University, 1020 Vienna, Austria

**Keywords:** euthanasia, end-of-life decisions, the dying patient act, palliative care, ethics, health policy

## Abstract

The demand for medical assistance in dying remains high and controversial with a large knowledge gap to support optimal patient care. The study aimed to explore physicians’ attitudes regarding euthanasia and examine the factors that related to these attitudes. We surveyed 135 physicians working at a tertiary-care hospital in Israel. The questionnaire was comprised of demographic and background information, DNR procedure information, encounters with terminally ill patients, familiarity with the law regarding end-of-life questions, and Attitudes toward Euthanasia. About 61% agreed that a person has the right to decide whether to expedite their own death, 54% agreed that euthanasia should be allowed, while 29% thought that physicians should preserve a patients’ life even when they expressed the wish to die. A negative statistically significant relationship was found between the level of religiosity and attitudes toward euthanasia. The physicians’ attitudes towards euthanasia are quite positive when compared to other countries. The data shows a conflict of values: the sacredness of human life versus the desire to alleviate patients’ suffering. The Coronavirus-19 outbreak reinforces the importance of supporting physicians’ efforts to provide ethical and empathic communication for terminally ill patients. Future studies should aim to improve our understanding and treatment of the specific types of suffering that lead to end-of-life requests.

## 1. Introduction

### Background

Euthanasia-derived from a Greek term meaning “good death” refers to the intentional hastening of death of a patient by a physician with the intent of alleviating pain and suffering [1]. Its proponents focus on the respect of patient autonomy, self-determination, and forestalling suffering. Yet, many clinicians remain untrained in end-of-life processes, fearful of violating ethical and social norms and pointing to a slippery slope danger [2,3].

Euthanasia can be classified according to the role of the physician in the process. In passive euthanasia, the role of the physician is limited to suspending treatment or stopping extraordinary measures to prevent the prolongation of life. However, in active euthanasia, the physician takes deliberate steps to end the life of a person who has requested to end their suffering by administering a toxic substance that accelerates their death [4]. Active euthanasia is actively debated and widely rejected for ethical, religious, legal, and medical reasons. Physician-assisted suicide connotes the involvement of the physician in providing a lethal substance to a patient to self-administer painlessly [5]. It was criticized by some while it has been endorsed by a variety of countries. In some countries (e.g., The Netherlands, Belgium, Luxembourg, Switzerland, Colombia), it is legally possible for a physician to assist in ending a persons’ life under carefully proscribed conditions. In other countries (e.g., Germany, France, England, India, Israel) medical treatments may be withheld under certain conditions, but active euthanasia is strictly forbidden under all circumstances. Physician-assisted suicide is legal in nine US states and the District of Columbia (e.g., Hawaii, Maine, New Jersey, Oregon, Vermont, and Washington). The “Dying Patient Act” was passed in Israel in 2005. It prohibits active euthanasia but allows patients to give directives not to provide treatment at the end of their lives. The law also requires the health system to offer palliative care to dying patients. However, multiple barriers have been encountered in the implementation of the law, due to different interpretations and practices in the actual clinical setting. Many doctors have made it clear that helping a patient die is opposed by their values and professional goals [6].

Doctors avoid talking to patients about death and even avoid contact with terminally ill patients [7]. However, the public discourse has shifted in supporting a patient’s wishes in recent years from Do Not Resuscitate (DNR) to one of Allow Natural Death (AND) instruction [8]. Concerns have been raised regarding the interpretation of the constitutional right to life and the premise of ‘first do no harm’ [3]. Bentor et al. found that more than 70% of Israeli physicians believe that the patients have the right to decide whether to receive life-prolonging treatment and that physicians should have a candid conversation with their patients and ascertain their end-of-life wishes [9]. Existing attitudinal and social norm gaps between doctors and the public indicate a large gap in knowledge and ongoing social embarrassment related to end-of-life discussions [10].

The results of these surveys suggest that physicians are deeply polarized, with 43% of physicians maintaining they would recommend patients receive treatment or an experimental drug that may extend their life, and 65% believe that their patients’ lives should be saved despite their explicit wishes. Yet, about one-third (30%) of doctors believe that terminally ill patients receive unnecessary interventions. Karni et al. examined the attitudes of 2969 physicians toward euthanasia by giving them different case studies and inquired about how they would behave in each scenario [11]. They found that 55% of physicians were willing to assist a terminally ill patient who wanted to end their life where the medical condition justified it, while 31% of physicians were unwilling to support patients’ requests to die. However, in a US study, a high percentage of physicians indicated they would not prevent treatment, even if the patient requested assistance due to a lack of knowledge about their ethical and legal rights regarding end-of-life treatment decisions [12].

Whereas patients and their families can decide about end-of-life issues, physicians have a crucial role in the process. The physician’s attitudes and values are central in their guidance and support of a humane and ethical decision-making approach. Our study aims, in light of the ongoing knowledge gap regarding end-of-life support by physicians, to explore the attitudes of physicians regarding euthanasia and examine the factors that are related to these attitudes. The specific objectives were to examine the relationships between seniority, encountered terminally ill patients in their work and/or personal life, religiosity, and attitudes toward euthanasia. In addition, to examine the differences between gender, religion, and specialty in relation to their attitudes.

## 2. Materials and Methods

### 2.1. Study Design

We conducted a cross-sectional survey in this study. The study was conducted by distributing questionnaires to physicians working at Barzilai University Medical Center, Israel, during January–February 2019. Barzilai University Medical Center is a 600-bed hospital located in southern Israel, serving a population of about 500,000, with more than 100,000 admissions annually. The study received approval from the Ashkelon Academic College Ethics Committee and the hospital leadership.

### 2.2. Participants and Procedure

A convenience sample of 135 physicians participated in the survey of 230 questionnaires distributed to all physicians working on the hospital wards (59% response rate). Questionnaires were distributed to physicians present on the wards during the study and that consented to answer the survey. A hard copy was given to each participant with an envelope addressed to their respective departments’ secretaries and were asked to return the completed questionnaire the next day in an envelope to maintain anonymity). The questionnaire included a cover letter describing the study and a consent form. The questionnaire took an average of 10 min to complete. The reasons for refusing to complete the questionnaire were given as time constraints and/or, heavy workload.

### 2.3. The Survey Questionnaire

A survey was provided for anonymous completion. The questionnaire was piloted and validated with two bio-ethicists experts, and their comments were integrated into the questionnaire. As part of the pilot, the questionnaire was distributed to 30 physicians working at the hospital, and the internal reliability of the questionnaire was tested (Cronbach’s α = 0.86). We also asked them to write comments on the questionnaire if there is an item that is not clear, adjusting accordingly. It was comprised of 29 closed-ended questions as follows:

- Demographic and background information—gender, age, marital status, religion, intrinsic religiosity [13], country of birth, country where studied medicine, seniority since graduation from medical school, the field of specialization;
- DNR Procedure—Does a DNR (Do Not Resuscitate) procedure exist in your department, to what extent does the dilemma of whether to order DNR exist, the extent to which medical teams have to decide whether to order a DNR;
- Encounters with terminally ill patients—Have you encountered terminally ill patients during work or personal life on a scale ranging from 1 (“1 = not at all”) to 5 (“5 = to a great extent”) with an option to mark “irrelevant”;
- Familiarity with the law regarding end of life questions—on a scale ranging from 1 (“1 = not at all”) to 5 (“5 = to a great extent”) with an option to mark “irrelevant”; and
- Attitudes toward Euthanasia—12 questions adapted from Bentor et al. [9]. The participants were asked to mark their agreement on each statement on a scale ranging from 1 (“strongly disagree”) to 5 (“strongly agree”), with an option to mark “irrelevant”. Using a confirmatory factor analysis, the questionnaire was divided into two dimensions: attitudes toward assisted passive/active euthanasia (for example: “doctors must consent to the patient’s request to prevent or terminate life-preserving treatment”) and attitudes toward autonomy for patient/family autonomy (for example: “an individual has the right to decide whether to expedite his death). The questionnaire’s internal reliability was Cronbachs’ α = 0.84. The “attitudes toward assisted passive/active euthanasia” dimension internal reliability was Cronbachs’ α = 0.81. The “attitudes toward autonomy for patient/family autonomy” dimension internal reliability was Cronbachs’ α = 0.88. The survey was designed specifically for this study and is included in Supplementary Materials for reference.

### 2.4. Data Analysis

The exploratory data analysis demonstrated that the data were normally distributed, and parametric statistical tests were examined by calculating Pearson correlations. The alpha coefficients of each of the scales were computed to measure the questionnaire’s internal reliability. The differences between the groups of physicians were analyzed using independent *t*-tests, χ^2^ tests, and one-way Analysis of Variance (ANOVA) according to the variables’ measurement scale. The results of the post-hoc evaluation were calculated by using Scheffe’s method [14]. A (multiple) linear regression model was used to test the multivariate prediction of attitudes toward euthanasia (stepwise backward). All reported *p* values are based on two-sided tests and were considered significant when below 0.05. All statistical analyses were performed using SPSS v.26 (IBM, Armonk, NY, USA).

## 3. Results

### 3.1. Respondent Demographics

A total of 135 physicians were included in the study. All worked at the Barzilai University Medical Center, Israel. Table 1 shows that among the respondents, there were no statistically significant differences in age, gender, and specialization between the groups. The mean range age of the respondents was 42 ± 12.54 years. In terms of religion, differences were found between the groups, with 83% of specialists being Jewish, and 59% of residents (and 53% of interns). There were also significant differences in the level of religiosity, with 89% of specialists defining themselves as secular, compared to 65% of residents and 50% of interns. Forty-one percent of the specialists were born in Israel and 20% studied in Israel, 74% of the residents were born in Israel but only 29% studied in Israel, and all the interns were born in Israel, but less than half studied in Israel (47%). The data reflect physicians working in peripheral hospitals, with more non-Jewish residents and interns, and who generally did their medical training outside of Israel, as described by Ashkenazi et al. [15].

### 3.2. Attitudes toward Eeuthanasia

The distribution of responses in regard to the attitudes toward euthanasia, after grouping categories, were as follows: answers 1+2 “agree slightly”, answer 3 remains “Agree moderately”, answers 4+5 “Strongly agree”. The distribution presented in Table 2.

After reversing the scales for ease in interpreting the data in the opposing questions, the mean’s of the relevant questions, as shown in the table, was calculated for each participant. The average of attitudes toward assisted passive/active euthanasia was 3.35 ± 0.79, the mean for attitudes toward autonomy for patient/family members was 3.76 ± 0.83, and the overall average for attitudes was 3.53 ± 0.72.

### 3.3. DNR Procedure, Familiarity with the “Dying Patient Act” and Role of Previous Encounter Terminally Ill Patients

A fifth (20%) of the doctors answered “yes”, 39% answered “no”, and the rest did not know (41%) if there is a DNR (Do Not Resuscitate) procedure in their hospital. The distribution of responses to the statements dealing with the dilemma of applying the DNR procedure, familiarity with the law, and the degree of encountering terminally ill patients are presented in Table 3.

### 3.4. The Relationship between Background Factors and Attitudes toward Euthanasia

Table 4 highlights the differences between the groups’ attitudes towards euthanasia. The data demonstrate that women expressed more positive attitudes toward patient autonomy, and Jewish physicians have more positive attitudes toward euthanasia and toward patient autonomy in relation to non-Jewish physicians. Internal medicine-trained physicians hold the most positive attitudes toward euthanasia and patient autonomy, followed by surgical specialists, pediatric, and finally diagnostic professions. A follow-up Scheffe test showed that diagnostic specialists held significantly more negative positions in relation to positions espoused by internal medicine and surgical specialists.

Testing the relationship between the factors revealed that the more senior the physicians, the more their attitudes were significantly positive (r_p_ = 0.18, *p* *=* 0.04); similarly, when they encountered more terminally ill patients in their work and/or personal life (r_p_ = 0.17, *p* = 0.04; r_p_ = 0.35, *p* < 0.001, respectively). A negative statistically significant relationship was found between the level of religiosity and attitudes toward euthanasia (r_p_ = −0.43, *p* < 0.001).

### 3.5. Linear Regression Model to Predict Attitudes toward Euthanasia

We used linear regression analyses to assess the comparative importance of variables in determining attitudes. The results of the multiple linear regression model to predict attitudes toward euthanasia are presented in Table 5. The models included variables that were significantly predictive models related to the attitudes in the univariate analyses. Table 5 demonstrates information about the direction of associations between variables and information about the different categories of variables, with a significant regression obtained (F_(126)_ = 17.45, *p* < 0.001), with an explained variance of 42%. All five predictors were significant contributors, with the level of religiosity the best predictor of attitudes toward euthanasia (*β* = −0.42, *p* < 0.001), with the more religious the doctor, the more negative the attitudes towards end-of-life care. It was followed by religion, with Jewish physicians having a more positive attitude (*β* = −0.22, *p* = 0.008), a familiarity with the law (*β* = 0.22, *p* = 0.005), country of birth (*β* = −0.18, *p* = 0.02), and previously encountering terminally ill patients at work (*β* = 0.17, *p* = 0.02).

## 4. Discussion

The physicians’ attitudes in Israel towards euthanasia are quite positive when compared to other countries, such as U.K. [16], France [17], Italy [18], Finland [19], Greece [20]. The statements with the highest degree of consent were those related to supporting decision-making by the patient or by family members. Such support might lower the emotional burden and responsibility of the physician regarding the patient’s end-of-the-life request. This finding points to the importance of the discussion with the patient and their family about the quality of the terminally ill patient’s life and the role of the family in supporting palliative options for the patient. It also raises ethical questions about whether a physician can refuse a terminally ill patient and/or their family’s request for help when the patient faces intolerable suffering and pain.

We found that 53% of physicians disagreed with the statement “*In any situation, the doctor should preserve the patient’s life, even if he wishes for an expedited death,*” while Bentor et al. [9] found that 56% of physicians believed that patients’ lives should be saved in any situation despite the patient’s request. Farber et al. [12] also found that a high percentage of physicians would not have prevented treatment even if the patient had requested it. In the current study, most doctors think that treatments of terminally ill patients are not unnecessary, and in a situation where a patient suffers from severe pain, taking a lethal dose should not be allowed. At the same time, the majority of physicians agreed that a patient has the right to decide to expedite their death, that a DNR procedure should be considered only when the treatment team thinks that resuscitation is unnecessary, and don’t agree with the statement that disconnecting a patient in a coma from resuscitation/ventilation machines is immoral.

These findings indicate the daily dilemmas physicians face when on the one hand, they are committed to protecting the sanctity of life, and yet, on the other hand, they are committed to alleviating the patient’s suffering while respecting their autonomy and choice. We found no differences between men and women regarding attitudes toward euthanasia which is in line with other studies [21,22]. As for the positive relationship between seniority and attitudes toward euthanasia, previous studies have found that euthanasia is more favorable among older, veteran physicians who have had previous encounters with terminal patients and are more likely to provide patients with lethal drugs if asked to by terminal patients seeking to end their lives [21,23].

The negative relationship between the level of religiosity and attitudes has also been found in many studies [24,25,26,27]. We know that physicians with different specializations have different attitudes towards euthanasia. For example, oncologists receive many more euthanasia requests and are more willing to provide end-of-life assistance than other physicians [28]. Geriatricians, in another study, had the highest frequency of caring for patients requiring end-of-life supportive care, in contrast to cardiologists, where the frequency was less than one percent [29].

The strengths of our study include a good response rate from a broad range of specialties and physicians’ status (specialist, residents, interns), as well as both Jewish and Arab physicians. Moreover, we used a validated attitudes questionnaire and that demonstrated that the level of religiosity was found to be the most strongly predictive about attitudes toward euthanasia. The regression model produced significant predictive models regarding the following factors, including the level of religiosity, religion, familiarity with the Dying Patient Act, country of birth, and past experiences encountering terminally ill patients’ attitudes toward euthanasia.

This article has several important limitations to consider. First, focusing on clinician perceptions relies on self-reports of current and past events, which may be a source of richness but also a source of bias. These perceptions could not be independently verified. Second, the sample was quite limited and unlikely to have equal representation from all departments and specialties at one sampling point in time. Third, the data represent only one major teaching hospital, which limits the generalizability of our findings. Fourth, because of the workload and the sensitive nature of this topic, we had difficulty recruiting physicians to complete the questionnaire and likely discouraged some participants from responding despite elaborate efforts to protect their anonymity. Fifth, we cannot tell if there are significant differences between the survey responders and the non-responders. Because of the anonymity of the subjects, nonrespondents could not be contacted for follow-up.

The implications from our study demonstrate the feasibility and importance of using multi-variable models to understand the deep and complex social attitudes towards end-of-life care decisions. The use of longitudinal study designs that track variations in attitudes through, and beyond, training, should offer an ideal design to fully understand how and why more positive attitudes develop within healthcare professionals. Further work is required to replicate these findings and explore qualitatively whether, and how, opinions of more religious or from other ethnicities might affect the treatment choices of terminally ill patients in general, and their attitudes towards euthanasia, in particular. Further research is needed that combines in-depth interviews with policymakers, physicians, patients, and family members to more deeply understand the experiences and attitudes of all parties.

We would be remiss if we did not position these findings in the context of the unprecedented end-of-life questions that have arisen in the past 10 months due to the Coronavirus (COVID-19) outbreak. As of mid-May, 3,339,000 people have died from COVID-19 across the globe (WHO COVID-19 Dashboard) [30]. The unprecedented global situation has forced health care providers across the world to consider end-of-life issues in the face of finite critical care support such as staff, beds, and equipment are necessary now more than ever [31]. Preparation for an impending death through end-of-life discussions and human presence when a person is dying is important for both patients and families. Pandemic planning must encompass the wider issues of deciding who to treat and who should not be treated and how to prepare physicians for these new emotional burdens. The findings of this study on attitudes of physicians and related factors underscore the importance of how to act in the Covid-19 pandemic. Clear and timely communication with the patient and their care is essential. Conveying hope that treatments will help needs to be sensitively balanced with an explicit acknowledgment that patients are sick enough to die [32]. Dying from COVID-19 negatively affects the possibility of holding end-of-life discussions because of social distancing and restrictions on visits [33]. Of related concern, recent reports have suggested that the Do-not-resuscitate orders in the U.K. were wrongly allocated to some care home residents during the Covid-19 pandemic, causing potentially avoidable deaths [34]. Compelling end-of-life decisions in these challenging times reinforces the urgency to act based on our study’s findings and raises the importance of supporting physicians in their efforts to provide ethical and empathic communication for terminally ill patients.

## 5. Conclusions

The medical ethics considerations surrounding euthanasia remain a global and controversial concern. It is important to bring the euthanasia discourse onto the public agenda, consider the sentiments of patients, families, doctors, including also religious and legal considerations, both as presented by the various stakeholders, and inviting professionals from these fields. Our findings contribute to a deeper understanding of the importance of physician opinions and corroborate international opinions on the thorny issues of end-of-life care. Physicians should be provided with the professional and emotional tools to deal with the dilemmas they experience during their work with terminal patients. At the same time, the growing trend toward legalization of assisting patients in their end-of-life requests in many parts of the world should prompt the healthcare and research community to improve our understanding and treatment of terminally ill and suffering patients. It also reminds us of the need to consider new guidelines that support co-design of care with family involvement during the late stages of terminal illnesses.

Physicians should be informed about DNR procedures, both within national and local institutional contexts, and efforts are needed to educate physicians about end-of-life legislation such as the Israeli Dying Patient Act. There is a need for well-designed curricula on palliative care and pain management within medical schools and residency programs to help trainees better reflect on experiences with end-of-life care and how best to support dying and suffering patients. Senior faculty need to appreciate the importance of sharing their experiences with, and reflections about euthanasia and end-of-life treatment dilemmas and how they have learned to make the most sense of them. This can instill a more professional and emphatic approach toward terminal care in future physicians.

## Figures and Tables

**Table 1:** Physician Respondents Characteristics.

Character	Sample (*n* = 135)		Specialist (*n* = 76, 57%)		Residents (*n* = 27, 19%)		Interns (*n* = 32, 24%)		χ^2^/F
	n	%	n	%	n	%	n	%	
Men	97	72	52	68	19	70	26	81	NS
Female	38	28	24	32	8	30	6	19	
In relationship	103	76	57	75	26	96	20	63	χ^2^ = 9.41 **, *p =* 0.009
Jewish	96	71	63	83	16	59	17	53	χ^2^ = 12.02 **, *p =* 0.002
Religiosity:									χ^2^ = 25.91 ***, *p* < 0.001
Secular	101	75	68	89	17	65	16	50	
Traditional	24	18	6	8	5	18	13	41	
Religious	10	7	2	3	5	18	3	9	
Born in Israel	83	62	31	41	20	74	32	100	χ^2^ = 4.86 ***, *p* < 0.001
Studied in Israel	36	28	15	20	7	29	14	47	χ^2^ = 7.60 *, *p* = 0.02
Specialization:							-	-	NS
(without interns)									
Surgical	35	35	29	38	6	24			
Internal	36	36	23	30	13	52			
Diagnostic	11	11	9	12	2	8			
Pediatrics	19	18	15	20	4	16			
Age (M ± SD) Range: 24–73	42 ± 12.54		50 ± 10.17		34 ± 6.10		29 ± 3.81		F = 85.51 ***, *p* < 0.001
Seniority (M ± SD) Range: 0.5–50	16 ± 13.08		23 ± 10.66		6 ± 3.66		1 ± 0.44		F = 95.56 ***, *p* < 0.001

* *p* < 0.05, ** *p* < 0.01, *** *p* < 0.001.

**Table 2:** Attitudes toward Euthanasia and Patient Autonomy (*n* = 135).

Statement	Slightly (%)	Moderately (%)	Strongly (%)	Irrelevant (%)	Mean ± SD **
**Attitudes toward assisted passive/active euthanasia (Advocates euthanasia)**					
Doctors must consent to the patient’s request to prevent or terminate life-preserving treatment	15	27	56	2	3.63 ± 1.15
* In any situation, the doctor should preserve the patient’s life, even if he wishes for an expedited death	53	14	29	4	1.60 ± 1.46
If a terminally ill patient suffers unbearably and is unable to make decisions, giving the patient a lethal dose of treatment should be allowed	46	15	28	11	2.54 ± 1.45
* Disconnecting CPR machines from a patient suffering from a coma is immoral	40	24	31	5	1.84 ± 1.39
If a patient is terminally ill, then he will be interested in euthanasia	14	25	53	8	3.69 ± 1.31
If a patient receives a DNR order, does the medical staff believe that the patient’s treatment is fruitless?	32	18	49	11	3.10 ± 1.50
To what extend is this true: “At the end of one’s life, it is better to end suffering than to preserve life?”	12	18	67	3	3.95 ± 1.15
**Attitudes toward autonomy for patient/family members (Advocates autonomy)**					
If a patient is unable to make decisions, his relatives should be allowed to decide whether to maintain life-preserving therapy	34	29	33	4	2.95 ± 1.24
An individual has the right to decide whether to expedite his death	15	19	61	5	3.80 ± 1.31
Euthanasia should be allowed for any individual who requests it	18	23	54	5	3.56 ± 1.30
An individual must fill a preliminary instruction regarding his wishes in a terminal situation	11	13	73	2	4.02 ± 1.13
Doctors must include the patient and his family in making an end-of-life decision	3	10	87	0	4.51 ± 0.82

* Opposite items; the data are presented before inversion of scales. ** The average is calculated excluding the option “irrelevant”.

**Table 3:** Distribution of Responses about DNR procedure, Familiarity with the “Dying Patient Act” and Previous Encounters with Terminally Ill Patients (*n* = 135).

Statement	Weakly (%)	Moderately (%)	Strongly (%)	Irrelevant (%)	Mean ± SD *
To what extent have you dealt with the dilemma of dealing with a DNR order	43	18	23	16	2.56 ± 1.30
To what extent is there a conflicting feeling in medical teams to order DNR	28	27	29	16	3.00 ± 1.07
How thoroughly informed are you about the “Dying Patient Act”	27	21	50	2	3.30 ± 1.29
To what extent have you encountered terminally ill patients in the professional setting	42	24	34	-	2.97 ± 1.27
To what extent have you encountered terminally ill patients in the personal setting	52	30	18	-	2.61 ± 1.04

* The average is calculated excluding the option “irrelevant”.

**Table 4:** Differences between Gender, Religion, and Specialty Regarding Attitudes towards Euthanasia.

	Variables	Categories	N	Mean ± SD	*t/F*	*p*
Gender	Advocates euthanasia	men women	97 38	3.35 ± 0.81 3.36 ± 0.75	0.10	0.92
	Advocates autonomy	men women	97 38	3.63 ± 0.79 4.06 ± 0.86	2.71	0.008
	General attitudes	men women	97 38	3.47 ± 0.72 3.67 ± 0.71	0.10	0.15
Religion	Advocates euthanasia	Jewish Non-Jewish	96 39	3.54 ± 0.80 2.89 ± 0.58	5.19	<0.000
	Advocates autonomy	Jewish Non-Jewish	96 39	3.97 ± 0.73 3.23 ± 0.84	5.04	<0.000
	General attitudes	Jewish Non-Jewish	96 39	3.73 ± 0.68 3.03 ± 0.57	5.55	<0.000
Specialty (without interns)	Advocates euthanasia	Internal Surgical Pediatrics Diagnostic	36 35 19 11	3.64 ± 0.77 3.41 ± 0.83 3.22 ± 0.83 2.56 ± 0.51	5.07	0.003
	Advocates autonomy	Internal Surgical Pediatrics Diagnostic	36 35 19 11	3.82 ± 0.76 3.81 ± 0.78 3.79 ± 0.95 2.98 ± 0.83	3.40	0.02
	General attitudes	Internal Surgical Pediatrics Diagnostic	36 35 19 11	3.71 ± 0.67 3.58 ± 0.72 3.49 ± 0.73 2.79 ± 0.57	5.05	0.003

**Table 5:** Linear Regression Model for Attitudes toward Euthanasia.

Variable	*β*	*B*	*p*
Religiosity	−0.42	−0.48	<0.000
Religion (0-Jewish)	−0.22	−0.33	0.008
Familiarity with the law	0.22	0.12	0.005
Country of birth (0-Israel)	−0.18	−0.26	0.02
Encountering terminally ill patients at work	0.17	0.20	0.02
R^2^	0.42		<0.000
Adj. R^2^	0.40		<0.000
*N*	135		
F_(df)_	18.26_(130)_		<0.000

## Data Availability

The data that support the findings of this study are available from the first author upon request.

## Supplementary Materials

The following are available online at https://www.mdpi.com/article/10.3390/ijerph18126396/s1, Supplementary File: A short survey about your attitudes regarding euthanasia.

## Abbreviations

COVID-19	coronavirus disease 2019
DNR	do not resuscitate
