# Supplementary material for: Facing Death: Attitudes toward Physician-Assisted End of Life among Physicians Working at a Tertiary-Care-Hospital in Israel
Source: Int J Environ Res Public Health. 2021 Jun 13;18(12):6396. doi: 10.3390/ijerph18126396 (PMC8296204; doi:10.3390/ijerph18126396)
Supplement: Supplementary file 1 [file ijerph-18-06396-s001.zip › ijerph-1242714-supplementary.pdf]

Dear participant,

Below is a short survey about your attitudes regarding euthanasia. The survey is confidential and anonymous, and completing it will take no more than five minutes. The findings will allow the formulation of recommendations for policy makers.

Please enter the complete questionnaire in the enclosed envelope and give it to the department secretary.

Thank you for your cooperation.

For more information, contact Dr. Keren Dopelt dopelt@bgu.ac.il

Please rate the degree to which you agree with the following statements:

	Strongly disagree (1)	2	3	4	Strongly agree (5)	Irrelevant (6)
1. Doctors must consent to the patient's request to prevent or terminate life-preserving treatment						
2. *In any situation, the doctor should preserve the patient's life, even if he wishes for an expedited death						
3. If a terminally ill patient suffers unbearably and is unable to make decisions, giving the patient a lethal dose of treatment should be allowed						
4. * Disconnecting CPR machines from a patient suffering from a coma is immoral						
5. If a patient is terminally ill, then he will be interested in euthanasia						
6. If a patient receives a DNR order, does the medical staff believe that the patient's treatment is fruitless?						
7. To what extend is this true: "At the end of one's life, it is better to end suffering than to preserve life?"						
8. If a patient is unable to make decisions, his relatives should be allowed to decide whether to maintain life-preserving therapy						
9. An individual has the right to decide whether to expedite his death						
10. Euthanasia should be allowed for any individual who requests it						
11. An individual must fill a preliminary instruction regarding his wishes in a terminal situation						
12. Doctors must include the patient and his family in making an end-of-life decision						

	Not at all (1)	2	3	4	To a great extent (5)	Irrelevant (6)
To what extent have you dealt with the dilemma of dealing a DNR order						
To what extent is there a conflicting feeling in medical teams to order DNR						
How thoroughly informed are you about the "Dying Patient Act"						
To what extent have you encountered terminally ill patients in the professional setting						
To what extent have you encountered terminally ill patients in the personal setting						

Some demographic and background questions:

1. Sex: 1. Man 2. Woman 3. I do not want to answer

2. Marital status: 1. In a relationship 2. I am not in a relationship

3. Do you have children? 1. Yes 2. No

4. Age: _______

5. Religion: 1. Jew 2. Muslim 3. Christian 4. Atheist 5. Other

6. Level of religiosity: 1. secular 2. traditional 3. religious

7. Country of birth: 1. Israel 2. Other: _____

8. Country where studied medicine: 1. Israel 2. Other: _____

9. Seniority since graduation from medical school: _____

10. field of specialization: 1. Internal 2. Surgical 3. Pediatrics 4. Diagnostic

11. Are you: 1. Specialist 2. Resident 3. Intern

12. Does a DNR (Do Not Resuscitate) procedure exist in your department? 1. Yes 2. No 3. I do not know
